# The Clinical Role of Serum Epidermal Growth Factor Receptor 3 in Hepatitis C Virus-Infected Patients with Early Hepatocellular Carcinoma

**DOI:** 10.3390/biology10030215

**Published:** 2021-03-11

**Authors:** Gian Paolo Caviglia, Maria Lorena Abate, Emanuela Rolle, Patrizia Carucci, Angelo Armandi, Chiara Rosso, Antonella Olivero, Davide Giuseppe Ribaldone, Francesco Tandoi, Giorgio Maria Saracco, Alessia Ciancio, Elisabetta Bugianesi, Silvia Gaia

**Affiliations:** 1 Department of Medical Sciences, University of Turin, 10100 Turin, Italy; marialorena.abate@unito.it (M.L.A.); angelo.armandi@unito.it (A.A.); chiara.rosso@unito.it (C.R.); antonella.olivero@unito.it (A.O.); davidegiuseppe.ribaldone@unito.it (D.G.R.); giorgiomaria.saracco@unito.it (G.M.S.); alessia.ciancio@unito.it (A.C.); elisabetta.bugianesi@unito.it (E.B.); 2 Division of Gastroenterology, Città della Salute e della Scienza University-Hospital, 10100 Turin, Italy; erolle@cittadellasalute.to.it (E.R.); pcarucci@cittadellasalute.to.it (P.C.); 3 Liver Transplant Unit, General Surgery 2U, Department of Surgical Sciences, Città della Salute e della Scienza University-Hospital, 10100 Turin, Italy; francesco.tandoi@gmail.com

**Keywords:** BCLC, biomarker, ERBB3, HCC

## Abstract

**Simple Summary:**

The identification of diagnostic and prognostic biomarkers for the management of patients with hepatocellular carcinoma is an unmet need. Here we investigate the potential clinical utility of the measurement of serum epidermal growth factor receptor 3 (i.e., ERBB3) in hepatitis C virus-infected patients with early hepatocellular carcinoma. Median values of serum ERBB3 were similar between patients with cirrhosis and those with early hepatocellular carcinoma (HCC); therefore, the measurement of the biomarker in the setting of HCC surveillance appeared unsuitable. Conversely, in patients with early HCC, serum ERBB3 values were significantly associated with overall survival, suggesting that the biomarker may be useful to tailor appropriate treatment strategies in hepatitis C virus (HCV)-infected patients with early hepatocellular carcinoma.

**Abstract:**

Epidermal growth factor receptor 3 (ERBB3) is a surface tyrosine kinase receptor belonging to the EGFR/ERBB family, involved in tumor development and progression. We evaluated the diagnostic and prognostic value of serum ERBB3 measurement in hepatitis C virus (HCV)-infected patients with early hepatocellular carcinoma (HCC). A total of 164 HCV-infected patients (82 with cirrhosis and 82 with early HCC) were included in the study. HCC was classified according to the Barcelona Clinic Liver Cancer (BCLC) staging system. Among patients with HCC, 23 (28%) had a diagnosis of very early tumor (BCLC = 0), while 59 (62%) had a diagnosis of early HCC (BCLC = A). Median overall survival (OS) in patients with HCC was 79.2 (95% CI 51.6–124.8) months. While ERBB3 serum values were similar between patients with cirrhosis and those with HCC (*p* = 0.993), in the latter, serum ERBB3 ≥ 2860 RU resulted significantly and independently associated with OS (Hazard Ratio = 2.24, 95% CI 1.16–4.35, *p* = 0.017). Consistently, the 1-, 3-, and 5-year OS rates in patients with serum ERBB3 ≥ 2860 RU were 90% (36/40), 53% (19/36), and 28% (8/29) in comparison to patients with serum ERBB3 < 2860 RU, which were 98% (40/41), 80% (32/40), and 74% (26/35) (Log-rank test; *p* = 0.014). In conclusion, serum ERBB3 values resulted an independent prognostic factor of patients with early HCC and might be useful to tailor more personalized treatment strategies.

## 1. Introduction

Hepatocellular carcinoma (HCC) accounts for 90% of all liver cancers and is one of the leading causes of death worldwide, with 841,000 new cases and 782,000 deaths per year [1]. Despite the availability of direct-acting antivirals, chronic hepatitis C virus (HCV) infection still represents a major cause of liver disease and cirrhosis, a condition at high risk for HCC development [2,3]. It has been calculated that 2.5% of patients affected by chronic HCV infection develop HCC [4], and the incidence rate of HCC is expected to further increase between 2018 and 2030 [5].

The prognosis of HCC varies greatly according to the tumor stage at diagnosis and overall liver function status; therefore, a timely detection of the tumor is crucial in order to allow curative treatments and thus to improve patients’ survival [6,7]. According to international guidelines, surveillance for HCC should be performed in patients at risk for tumor development by means of ultrasound (US), with or without the measurement of serum alpha-fetoprotein (AFP) [8,9], while the Barcelona Clinic Liver Cancer (BCLC) staging system should be adopted for HCC prognostication and treatment allocation [8,9]. However, US has sub-optimal sensitivity for early HCC detection [10,11]; therefore, there is a need for novel tools able to improve surveillance in patients at risk of HCC development. Furthermore, novel biomarkers are urgently needed in order to refine prognosis and to tailor personalized treatment strategies.

Epidermal growth factor receptor 3 (ERBB3) is a surface tyrosine kinase receptor belonging to the EGFR/ERBB family [12]. ERBB receptors are widely expressed in different cell types and play a pivotal role in many cellular functions, including cell growth, division, migration, adhesion, and apoptosis [13]. Moreover, increasing evidence supports the involvement of ERBB receptors, together with the process of epithelial–mesenchymal transition, in the development and progression of different types of human cancer [14,15,16,17]. Recent studies suggested that secreted ERBB3 isoforms were able to discriminate between patients with or without HCC [18], and that serum ERBB3 was associated with overall survival (OS) in patients with advanced HCC [19].

Here, we investigated the diagnostic and prognostic value of serum ERBB3 in HCV- infected patients with early HCC. 

## 2. Materials and Methods

### 2.1. Patients

This retrospective study included patients with HCV-related cirrhosis with or without a new diagnosis of HCC, recruited at the outpatient clinic of the Unit of Gastroenterology of Città della Salute e della Scienza di Torino—Molinette Hospital, Turin, Italy, between November 2012 and January 2018.

For all patients, the inclusion criteria were age ≥18 years, serum anti-HCV-positivity, diagnosis of cirrhosis, availability of a serum sample at the time of HCC diagnosis, and signed written informed consent. For patients with cirrhosis under surveillance, a minimum of 1-year US follow-up after the collection of serum sample was required. For patients with HCC, an additional inclusion criterion was a diagnosis of early tumor. 

The presence of cirrhosis was assessed by liver elastography (FibroScan^®^, Echosens™, Paris, France) or by hepatic ultrasound features and endoscopic signs of portal hypertension [20,21]. The diagnosis of HCC was achieved by histological examination or by contrast-enhanced imaging methods showing the radiological hallmark of HCC (i.e., the combination of hypervascularity in late arterial phase and washout on portal venous and/or delayed phases), following international guidelines [8]. Early HCC was classified according to the BCLC staging system (0 = very early; A = early) [8].

Serum samples were collected in polypropylene 2 mL tubes labeled with the study participant identification code and stored at −80 °C.

### 2.2. Measurement of Serum ERBB3

The HCC-REAAD™ ERBB3 ELISA kit (Restalyst Pte Ltd., Singapore) was used for the quantitative detection of human ERBB3 in serum. Briefly, the methods make use of two layers of antibodies (i.e., capture and detection antibodies) to determine patients’ ERBB3 protein levels in serum samples. An 11-point standard curve in 2-fold serial dilutions was used to quantify ERBB3 concentration in each sample. Optical density was red at 450 nm against a 620 nm reference filter. Final concentration was given in arbitrary REAAD™ Units (RU).

### 2.3. Statistical Analysis

Continuous variables were reported as median and interquartile range (IQR) while categorical variables were reported as number and percentages (%). Data normality was checked by the D’Agostino–Pearson test. The Mann–Whitney test and Fisher’s exact test were used to compare continuous and categorical variables, respectively. The Kruskal–Wallis test was used to compare continuous variables among more than two independent groups. Correlation between continuous variables was assessed by Spearman’s rank correlation coefficient (*r_s_*). Diagnostic accuracy was assessed by receiver operating characteristic (ROC) curve analysis reporting the area under the curve (AUC) value as estimate of accuracy. The survival curves were analyzed by the Kaplan–Meier method with the log-rank test. Multivariate Cox proportional-hazard regression analysis was performed to evaluate whether variables associated with OS at univariate analysis were independently associated with patient survival (Hazard ratio; HR).

All statistical analyses were performed by using MedCalc^®^ v.18.9.1 (MedCalc Software Ltd., Ostend, Belgium) and a *p* value smaller than 0.05 was considered statistically significant.

## 3. Results

A total of 164 patients (82 patients with HCC and 82 patients with cirrhosis without HCC) were included in the study. The demographic, clinical, and biochemical characteristics of the study population is reported in Table 1.

All patients were Caucasians and were all chronically infected with HCV. The 82 patients with HCC were significantly older than the 82 patients with cirrhosis (68, range 45–89 years vs. 58, range 49–82 years; *p* < 0.001). The ratio of males to total patients was 79% (65/82) in patients with HCC and 63% (52/82) in those with cirrhosis (*p* = 0.038). Patients with HCC showed worse liver function: higher alanine aminotransferase (ALT) (*p* = 0.019), higher aspartate aminotransferase (AST) (*p* < 0.001), lower platelet count (*p* < 0.001), lower albumin (*p* < 0.001), higher international normalized ratio (INR) (*p* = 0.007), and higher total bilirubin (*p* = 0.010) compared to patients with cirrhosis. Among patients with HCC, 23 (28%) had a diagnosis of very early tumor (BCLC = 0) while 59 (62%) had a diagnosis of early HCC (BCLC = A). As first-line therapy, 74 (90%) patients with HCC were treated with thermoablative percutaneous techniques; 65 with radiofrequency ablation (RFA) and 9 with microwave ablation (MWA) associated or not with percutaneous ethanol injection. Only 8 (10%) patients underwent surgery as curative treatment.

### 3.1. Diagnostic Accuracy of Serum ERBB3 for the Detection of HCC

**Figure 1:**
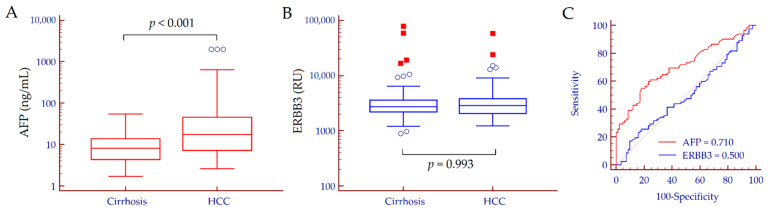
AFP (**A**) and ERBB3 (**B**) serum values in patients with cirrhosis and those with HCC and the corresponding receiver operating characteristic (ROC) curves (**C**). Hollow circles indicate values that are smaller than the lower quartile minus 1.5 times the interquartile range, or larger than the upper quartile plus 1.5 times the interquartile range; red squares indicate values that are smaller than the lower quartile minus 3 times the interquartile range, or larger than the upper quartile plus 3 times the interquartile range. *p* values were calculated by the Mann–Whitney test. Abbreviations: alpha-fetoprotein (AFP), area under the curve (AUC), epidermal growth factor receptor 3 (ERBB3), arbitrary REAAD™ Units (RU).

The performance of serum ERBB3 was compared to that of AFP as a reference biomarker for HCC detection. Median AFP values were significantly different between patients with cirrhosis and those with HCC (*p* < 0.001) (Figure 1A). Conversely, no difference was observed for serum ERBB3; in patients with cirrhosis, median ERBB3 values were 2736 (2213–3606) RU while in patients with HCC, they were 2859 (2064–3809) RU (*p* = 0.993) (Figure 1B). Consistently, AFP showed an AUC value of 0.710, 95% CI 0.634–0.778 (*p* < 0.001) for the discrimination between cirrhosis and HCC, while serum ERBB3 was not able to discriminate between the two groups of patients (AUC = 0.500, 95% CI 0.421–0.579, *p* = 0.994) (Figure 1C).

**Figure 2:**
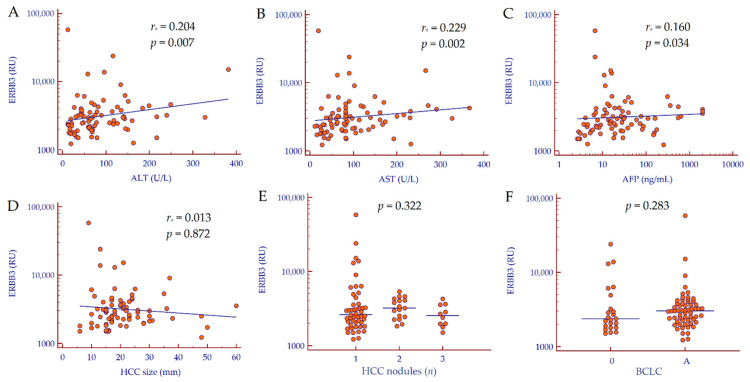
Correlation analysis. (**A**–**D**) Correlation between continuous variables was assessed by Spearman’s rank correlation coefficient. (**E**) *p* value was calculated by the Kruskal–Wallis test. (**F**) *p* value was calculated by the Mann–Whitney test. Abbreviations: alpha-fetoprotein (AFP), alanine aminotransferase (ALT), aspartate aminotransferase (AST), Barcelona Clinic Liver Cancer (BCLC), epidermal growth factor receptor 3 (ERBB3), hepatocellular carcinoma (HCC), number (*n*), arbitrary REAAD™ Units (RU).

In patients with HCC, serum ERBB3 values were moderately correlated to ALT (*r_s_* = 0.204, 95% CI 0.030–0.328, *p* = 0.007), to AST (*r_s_* = 0.229, 95% CI 0.053–0.352, *p* = 0.002), and to AFP (*r_s_* = 0.160, 95% CI 0.015–0.304, *p* = 0.034). Conversely, no correlation was observed between serum ERBB3 levels and tumor features (Figure 2).

### 3.2. Prediction of Overall Survival

The median follow-up of the 82 patients with HCC was 46.1 (95% CI 37.4–60.4) months, while the median OS was 79.2 (95% CI 51.6–124.8) months. Thirty-seven (45%) patients died during the follow-up period. For Kaplan–Meyer analysis, ERBB3 values were dichotomized according to the observed median concentration in the cohort of patients with HCC (i.e., 2859 RU).

**Figure 3:**
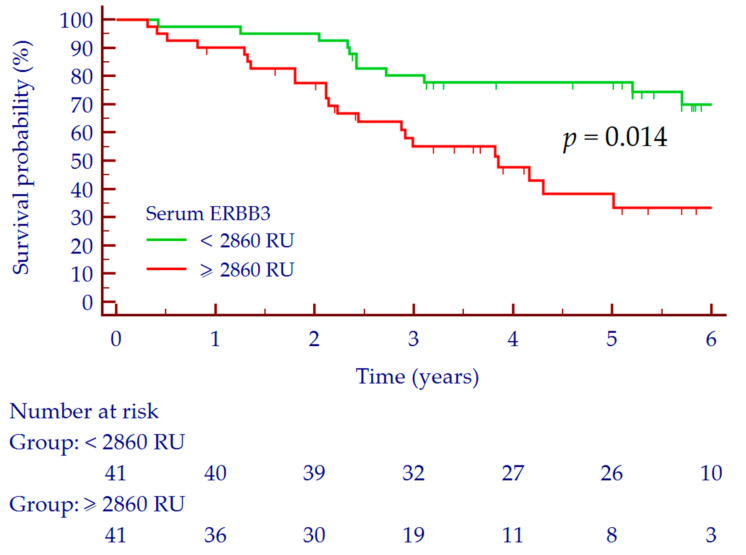
Survival curves according to serum ERBB3 ≥ 2860 RU. Small vertical lines indicate censored data. Abbreviations: epidermal growth factor receptor 3 (ERBB3), arbitrary REAAD™ Units (RU).

In the overall group of patients with HCC (*n* = 82), the 1-year survival rate was 95% (77/81), the 3-year survival rate was 67% (51/76) and the 5-year survival rate was 53% (34/64). According to serum ERBB3 values, the 1-, 3-, and 5-year OS rates in patients with serum ERBB3 ≥ 2860 RU were 90% (36/40), 53% (19/36), and 28% (8/29) in comparison to patients with serum ERBB3 < 2860 RU, which were 98% (40/41), 80% (32/40), and 74% (26/35) (Log-rank test; *p* = 0.014) (Figure 3). The AUC values for the prediction of 1-, 3-, and 5-year OS were 0.697, 95% CI 0.585–0.795 (*p* = 0.056), 0.685, 95% CI 0.568–0.787 (*p* = 0.004) and 0.759, 95% CI 0.636–0.857 (*p* < 0.001), respectively.

At multivariate Cox regression analysis adjusted for age, liver function (Child–Pugh score) and tumor stage (BCLC), ERBB3 ≥ 2860 resulted significantly and independently associated with OS (HR = 2.24, 95% CI 1.16–4.35, *p* = 0.017) (Table 2).

## 4. Discussion

In the present study, we investigated the clinical role of serum ERBB3 measurement in patients with early HCC. Although serum ERBB3 was not able to discriminate between patients with cirrhosis and those with HCC, we observed a potential prognostic value for the prediction of OS. Remarkably, the association with patients’ outcome was independent from age, liver function, and HCC stage.

Secreted isoforms of ERBB3 have been described in different types of cancer [22,23]; to the best of our knowledge, only the study by Hsieh and colleagues investigated the accuracy of serum ERBB3 for the detection of HCC [18]. In contrast to our findings, the authors reported a good diagnostic accuracy for the discrimination between patients with or without HCC; furthermore, the diagnostic accuracy of ERBB3 for HCC detection was significantly higher compared to the performance of AFP. However, several aspects should be taken into consideration when interpreting these results. Firstly, our study was performed on patients of Caucasian ethnicity chronically infected with HCV, while the study by Hsieh et al. included Asian patients prevalently infected with hepatitis B virus; in fact, previous reports showed that etiology of chronic liver disease could affect the performance of HCC biomarkers [24]. In addition, this is in good agreement with the results of a recent study showing that HBV and HCV infections were associated with different ERBB3 mRNA expression in HCC cells [25]. Regarding ethnicity, several studies showed that ethnic differences may influence cancer biomarker levels as well as drug response and survival, possibly reflecting different genetic alterations and epigenetic changes involved in the regulation of oncogenic pathways [26]. Secondly, patients with HCC included in the study by Hsieh et al. showed different tumor stages, ranging from early (solitary nodule, ≤2 cm, no vascular invasion and no metastasis) to advanced tumor (extrahepatic invasion and/or metastasis), while in our study we included only patients with early HCC. Furthermore, in our study we included as control group patients with liver cirrhosis only; as a matter of fact, cirrhosis is a pre-neoplastic condition characterized by genetic alterations, epigenetic modifications, and dysregulation in numerous signaling pathways involved in cellular growth, differentiation, and angiogenesis not yet established in patients with chronic hepatitis without cirrhosis [27,28]. These molecular alterations may result in an increased ERBB3 expression in cirrhotic patients compared to those with chronic hepatitis. Consistently, Hsieh et al. observed significantly higher ERBB3 serum values in the former group of patients compared to the latter [18]. Finally, the different assays used for ERBB3 measurement may be a not-negligible source of variation. Taken together, these major differences concerning inclusion criteria and methodological approach may explain the discrepancies between the results of the present study and the study by Hsieh et al.

Conversely, in the setting of HCC prognosis, the measurement of serum ERBB3 showed noteworthy results. Several studies already reported an association between ERBB3 expression and patients’ outcome in different types of cancer, including lung adenocarcinoma, breast, gastric, and colorectal cancer [29,30,31]. Consistently, it has been shown that the ERBB3-PI3K-Akt signaling pathway is directly involved in HCC growth, invasion, and migration [32]. Furthermore, ERBB3 was identified as a compensatory survival factor that was up-regulated after c-Met inhibition in c-Met^+^ HCC cell line [33]. In the present study, we observed that low values of serum ERBB3 resulted associated with longer OS; interestingly, the association remained significant even after adjustment for patients’ age, liver function, and tumor stage (HR = 2.24, *p* = 0.017). Similarly, Giannelli and colleagues previously showed that higher baseline plasma ERBB3 levels were significantly associated with poor survival (HR = 2.21, *p* < 0.001) in patients with advanced HCC [19].

For decades, treatment allocation followed a “stage hierarchy” approach, consisting in the selection of a specific treatment according to the HCC stage [34]. However, growing evidence supports a less rigid therapeutic approach based on the treatment stage migration concept [35], or on a hierarchical selection of therapy based on the respective proven effectiveness, independent from a specific HCC staging system [36]. In this regard, the results of our study may be valuable for the personalization of therapeutic strategies; as stated by the European Association for the Study of Liver Disease [8], the identification of novel biomarkers able to predict therapy response and recapitulate OS are an unmet need. Further prospective studies are needed to evaluate whether the measurement of serum ERBB3 could improve the selection of the most appropriate treatment for each patient with a diagnosis of HCC.

The present study might be limited by the size of the population included. Therefore, we were not able to stratify survival according to multiple serum ERBB3 cut-offs. However, as regards HCC patients, we deliberately included a homogeneous group of patients with early tumor to avoid possible bias. Furthermore, the results were corrected for potential confounding factors such as age, liver function, and tumor stage. Thus, we believe that the results are reasonably robust and highlight a potential clinical usefulness of the investigated biomarker.

## 5. Conclusions

Based on the results of the present study, the measurement of serum ERBB3 appeared unsuitable for the surveillance of patients with cirrhosis for the risk of HCC development; indeed, the biomarker was not able to discriminate between patients with cirrhosis and those with early tumor. However, circulating levels of ERBB3 resulted a significant prognostic factor able to predict the survival of HCV-infected patients with early HCC. Further studies are needed to evaluate whether serum ERBB3 might be useful to tailor personalized treatment strategies.

## Figures and Tables

**Table 1:** Characteristics of the patients included in the study according to hepatocellular carcinoma (HCC) diagnosis.

Characteristics	Cirrhosis	HCC	*p*-Value
Patients, *n*	82	82	
Age (years), median (range)	58 (49–82)	67 (45–89)	<0.001
Male gender, *n* (%)	52 (63%)	65 (79%)	0.038
ALT (U/L), median (IQR)	58 (33–89)	67 (36–126)	0.019
AST (U/L), median (IQR)	59 (31–73)	82 (47–132)	<0.001
Platelets (× 10^9^/L), median (IQR)	139 (88–187)	97 (68–124)	<0.001
Albumin (g/dL), median (IQR)	4.1 (3.8–4.4)	3.9 (3.3–4.1)	<0.001
INR, median (IQR)	1.07 (1.00–1.16)	1.15 (1.06–1.26)	0.007
Total Bilirubin (mg/dL), median (IQR)	0.8 (0.6–1.1)	1.0 (0.7–1.6)	0.010
AFP (ng/mL), median (IQR)	8.2 (4.4–14.0)	17.5 (7.3–46.0)	<0.001
Child–Pugh Score			
A, *n* (%)	80 (98%)	71 (87%)	0.018
B, *n* (%)	2 (2%)	11 (13%)	
BCLC Stage			
0, *n* (%)		23 (28%)	
A, *n* (%)		59 (62%)	
HCC nodules			
1, *n* (%)		51 (62%)	
2, *n* (%)		19 (23%)	
3, *n* (%)		12 (15%)	
Size of major nodule (mm), median (IQR)		18 (15–24)	

*p* values for quantitative variables were calculated by the Mann–Whitney test while *p* values for categorical variables were calculated by Fishers’ Exact test. Abbreviations: alanine aminotransferase (ALT), aspartate aminotransferase (AST), alpha-fetoprotein (AFP), Barcelona Clinic Liver Cancer (BCLC), hepatocellular carcinoma (HCC), international normalized ratio (INR), interquartile range (IQR), number (*n*).

**Table 2:** Multivariate Cox proportional-hazard regression analysis of the predictors of overall survival (OS).

Characteristics	HR, 95% CI	*p*-Value
Age, years	1.01, 0.98–1.05	0.370
Child–Pugh Score, B	0.77, 0.26–2.26	0.770
BCLC Score, A	1.40, 0.66–2.98	0.377
Serum ERBB3 ≥ 2860 RU	2.24, 1.16–4.35	0.017

Abbreviations: Barcelona Clinic Liver Cancer (BCLC), confidence interval (CI), hazard ratio (HR), epidermal growth factor receptor 3 (ERBB3), arbitrary REAAD™ Units (RU).

## Data Availability

The data presented in this study are available on request from the corresponding author.
